# Books Reviewed

**Published:** 1868-04

**Authors:** 


### Books Reviewed.

Spermatorrhœa: its Causes, Symptomology, Pathology, Diagnosis, Prognosis and Treatment. By Roberts Bartholow, A. M., M. D. Second edition, revised and enlarged. New York: Wm. Wood & Co., 61 Walker street, 1867.

The rapid exhaustion of the first edition within scarcely a year, is certainly evidence of the great attention which the author's views have elicited amongst medical men. Strongly opposed to Lallemand's conceptions of the pathology of this disease, but regarding it as essentially a *neuroses*, a functional derangement of the excito motor spinal nerves, the writer would limit the use of local applications of nitrate of silver to the prostatic portion of the urethra to those cases in which other less painful and dangerous practices are of no avail. The adoption of these views by the profession we believe is becoming more general, and we doubt not the work of Dr. Bartholow has largely been instrumental in their dissemination. We would bespeak for it a careful perusal of all those who may desire to investigate this malady as one of the very best, most careful and reliable treatises upon this subject.

Annual Abstract of Therapeutics, Materia Medica, Pharmacy and Toxicology for 1867; followed by an original Memoir on Gout, Gravel and Urinary Calculi. By A. Bouchardat, Professor of Hygiene to the Faculty of Medicine, Paris, etc. Translated and edited by M. J. De Rosset, M. D. Philadelphia: Lindsay & Blakiston, 1868.

The comprehensive title of this work conveys to the reader its character and scope. For many years this "Annual Abstract" has been published in Europe, and it is the favorable reception and the high estimation in which it is held that induced the translator to lay it before the profession of this country. The selections have been made with a special regard to the useful and practical information which they may convey, and from a careful examination of them we are fully prepared to endorse the hopes of the editor, that the work will meet "the requirements of physicians whose engagements do not permit of their searching over the immense field from which these fruits have been gathered, to practitioners in the country, as conveying the results of the active labors of the 'toilers' in our profession; and to medical men generally in the amount of original information from sources not hitherto available."

---

Studies in Pathology and Therapeutics. By Samuel H. Dickson, M. D., LL. D., Professor of the Practice of Physic in the Jefferson Medical College, Philadelphia, Pa., etc., etc. New York: Wm. Wood & Co , 61 Walker street, 1867.

From the introductory note we learn that four of the six essays contained in this volume have been delivered before a class in the Jefferson Medical College. These essays embrace a consideration of, 1st, disease, its charater and tendency; 2d, the causation of disease; 3d, of certain morbid conditions of the sensorial system; 4th, pneumonia; 5th, scrofulosis and tuberculosis; and 6th, therapeutics. The views which the author entertains upon these topics, are in many respects original and contrary to those generally accepted by the profession, yet they are logical deductions from his observations, and they will not fail to carry conviction and re-mould the opinions of many. In the essay (scrofulosis and tuberculosis) the author assumes the position that marriages of consanguinity do not predispose to scrofulosis or other degeneracy where both parties are free from constitutional disease, but that the physical and mental degeneracy so often met in marriages of consanguinity is to be attributed to an existing predisposition to this disease in a family. For the support of this view Dr. Dickson cites the prevalent customs of intermarriage among the Jews, the Arabs, etc., without any deterioration of these races, they formerly in fact being regarded as one of the best intellectually developed races, while the latter are physically considered athletic, energetic and active. Again; he does not regard the fair-haired and blonde-complexioned races as especially predisposed to this malady, but rather seeks for the causes in the climate, temperament and individual constitution. We could wish to present to our readers the author's views upon this and other subjects by more extensive citations would space permit it.

---

Plastics: a new Classification and brief Exposition of Plastic Surgery. By David Prince, M. D. Philadelphia: Lindsay & Blakiston, 1868.

This work is a carefully considered and eminently practical *resumé* of the existing state of the science and art of which it treats, presenting the whole subject in clear and concise language, thus making it especially adapted to the wants of physicians engaged in the active duties of their profession, who cannot well spare the time to read the more voluminous treatises upon these subjects. The author has adopted a new classification which he believes "will greatly aid in a better appreciation and application" of the principles of plastic surgery, which are included in the following six general methods:—1. Sliding in a direct line. 2. Sliding in a curved line. 3. Jumping. 4. Inversion or eversion. 5. Taliacotion; the part being obtained from a distance by grafting. The various operations the author describes by the recital of cases, pointing out the comparative value of the various operative procedures, and illustrating the whole subject with appropriate wood cuts.

Obstetrical Clinic; a Practical Contribution to the Study of Obstetrics and the Diseases of Women and Children. By George T. Elliot, M. D., etc., etc. New York: D. Appleton & Co., Broadway.

Dr. Elliot's fourteen years' service in Bellevue Hospital furnished the clinical experience embodied in this work. The cases which interested him most deeply, and which are believed to be most highly instructive to the profession, have been reported in detail, with such remarks as were made to the medical classes which were from time to time in attendance, together with such other practical hints as have suggested themselves during the preparation of the work.

This method of teaching obstetrics must prove exceedingly popular, really possessing advantages which will be apparent upon perusal. Reported cases of all the more difficult and perplexing diseases and accidents incident to the parturient state are embodied in the work, thus giving the student and practitioner a concise and practical guide to the symptoms, modes of treatment and termination of all, or nearly all these various conditions.

Many of the cases reported, have not been in Dr. Elliot's care alone, and we observe that in giving the details of treatment he has faithfully reported the facts, leaving us in ignorance, in some cases, whether the treatment adopted by his associates received his approval or not. Upon the 154th page, in the report of a very interesting case of *placenta previa*, where there had been great loss of blood, vomiting, sighing, etc., etc., with feeble pulse and many alarming symptoms, he says:

"June 5th, 10.30 A. M. Since the last note, Dr. F. has carefully fed the patient on beef-tea, brandy and opium, and she has rallied. Ergot kept in readiness, quinine and sul. acid given, and a blister has been applied over the abdomen, to anticipate metro-peritonitis."

We do not conclude that Dr. Elliot recommends a "blister to anticipate metro-peritonitis," or that he regards it as proper treatment even if it is present, but we fear that some physicians who believe in blistering every woman's belly which may appear tender or painful after labor, will infer that he approves of such treatment. It is by no means probable to us, that blistering would anticipate peritonitis, or relieve it in the slightest degree, if present, and we dislike to have any seeming countenance given to such plan of procedure by so distinguished an author and teacher.

In answering the question: "Should pelvic abscess from cellulitis always be opened promptly?" he says: "For my own part, now-a-days, as a rule, I rarely find it necessary to open mammary abscesses, abscesses in tonsellitis, and in cellulitis. Mammary abscesses must of course be opened, if their anatomical site, or the great vital tenacity of the skin, or very severe pain from tension, demand the operation; and abscesses in the deeper tissues of the throat may imperitively demand the knife; while if the pelvic abscess break into the peritoneal cavity one may well regret that the vaginal or rectal wall had not been previously punctured." "While I believe that this expectant plan is the best adapted as a rule, to secure good results in a large number of cases, I yet regard the decision as elective.' This quotatron is made for the benefit of conservative practice, and to show how careful our author has been of the opinions of others, and how fair and reasonable in the expression of his own.

As a guide in everything of which it treats, as a practical, candid, truthful and complete presentation of the present pathological and therapeutical history of the diseases and accidents incident to pregnancy and parturition, this work is unexcelled, unequaled in many respects. Clinical observations in obstetrics, reported with the greatest fidelity and faithfulness, as has been done by Doctor Elliot, cannot fail to interest every practitioner, and prove not only a guide in practice, but a standard by which the experience of others may be compared and their successes estimated.

---

Principles and Practice of Obstetrics. By Gunning S. Bedford, M. D. Fourth edition, carefully revised and enlarged. New York: William Wood & Co., 61 Walker street, 1868.

We are most happy to announce the appearance of the *fourth* edition of Prof. Bedford's work on Obstetrics. In the short period of six years, it has passed to its present and most complete edition, and has been everywhere received by the profession with unusual favor. When this work first made its appearance it was our privilege to speak in detail of its arrangement, contents, illustrations and other attractions, but it can now hardly be appropriate to mention its points of excellence; it is too well known to our readers and to the whole profession to make such notice acceptable.

Everything which can be said in favor of a woik upon Obstetrics has been already expressed in regard to this in its former editions; it now commends itself to still higher approval in its present revised and enlarged form. The subjects of anæsthetics and twin pregnancies have been dwelt upon more extensively in this than in former editions, and a lecture has also been added upon the complications of pregnancy, in which are discussed chorea, jaundice, paralysis, etc., etc. The illustrations so far as we observe remain unchanged; they could hardly be improved by change. Four colored lithographic plates and numerous wood cuts have added greatly to the value of the work, and make it much better fitted to the wants of both students and practitioners. Prof. Bedford has done a real service for the profession, and his work richly deserves the reputation it enjoys and the support it has so universalfy received.

